# A Modular Standard Operating Procedure for Standardizing Lithium Metal Interfaces

**DOI:** 10.1002/advs.76595

**Published:** 2026-07-15

**Authors:** Wen‐Hsin Chang, Anlin Shaju, Han‐Shiuan Lin, Sih‐Ling Hsu, Quang Huy Dinh, Che‐Ning Yeh, Elise Yu‐Tzu Li, Yu‐Sheng Su

**Affiliations:** ^1^ International College of Semiconductor Technology National Yang Ming Chiao Tung University Hsinchu Taiwan; ^2^ Department of Materials Science and Engineering National Tsing Hua University Hsinchu Taiwan; ^3^ Department of Chemistry National Taiwan Normal University Taipei Taiwan; ^4^ Industry Academia Innovation School National Yang Ming Chiao Tung University Hsinchu Taiwan

**Keywords:** artificial solid electrolyte interphase, chemical polishing, density functional theory, high‐energy‐density batteries, lithium fluoride, polycyclic aromatic hydrocarbons

## Abstract

Lithium metal is a promising material for high‐energy‐density batteries, yet its electrochemical behavior is strongly influenced by the poorly controlled initial surface state of commercial lithium foils. Variations arising from manufacturing, storage, and handling introduce interfacial heterogeneity, leading to performance scatter and limited reproducibility across studies. Herein, we establish a modular standard operating procedure (SOP) to systematically regulate lithium metal interfaces prior to testing. The SOP decomposes lithium pretreatment into three functionally distinct steps: chemical etching (E) to remove native surface layers, mechanical brushing (B) to homogenize surface geometry, and solution soaking (S) to induce a controlled artificial solid electrolyte interphase. Using symmetric Li||Li cells, the integrated E–B–S sequence exhibits improved voltage stability and suppressed interfacial resistance growth compared with partial or no treatments. Structural and interfacial analyses using SEM, lithium‐sensitive EDX, AFM, and EIS reveal the formation of a three‐dimensionally structured yet chemically uniform lithium surface. Density functional theory calculations and depth‐resolved XPS further clarify the chemical origins of controlled etching and interphase formation. The standardized lithium interface is validated in Li||LiFePO_4_ full cells, demonstrating enhanced capacity retention, reduced polarization, and improved rate capability. This work provides a transferable and reproducible framework for lithium metal interface standardization.

## Graphical abstract

A modular standard operating procedure (SOP) is developed to standardize lithium metal interfaces through sequential etching, brushing, and soaking. By transforming poorly controlled lithium surfaces into chemically uniform and structurally regulated interfaces, the framework improves electrochemical reproducibility, interfacial stability, and transferability across multiple battery configurations.

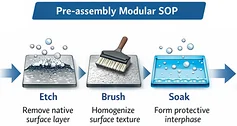

## 1 Introduction

Lithium metal is widely regarded as an ideal electrode material for next‐generation rechargeable batteries due to its ultrahigh theoretical capacity (3860 mAh g^−1^) and the lowest electrochemical potential among known metals [1, 2, 3]. These attributes make lithium metal particularly attractive for high‐energy‐density systems such as lithium–sulfur batteries, lithium–air batteries, and solid‐state lithium batteries [3, 4, 5, 6, 7]. Despite these advantages, the practical implementation of metallic lithium remains severely hindered by interfacial instability during repeated lithium plating and stripping [2, 8, 9]. In addition to serving as an active anode material, lithium metal is also ubiquitously employed as a counter and reference electrode in fundamental electrochemical studies, where its surface condition critically influences data reliability and comparability.

A critical yet often overlooked factor underlying this instability is the highly variable initial surface state of lithium metal. Commercial lithium foils are unavoidably affected by manufacturing processes, storage history, and handling conditions, leading to heterogeneous surface features such as rolling‐induced striations, native passivation layers, and uneven chemical contamination [10, 11]. Even when identical electrolytes, current densities, and cycling protocols are employed, such uncontrolled surface heterogeneity can introduce substantial performance scatter and complicate meaningful comparison across different studies. As a result, the electrochemical behavior attributed to electrolyte formulations or interfacial modifications may, in practice, be strongly convoluted with variations in the initial lithium surface condition. This issue is particularly critical when lithium metal is used as a reference electrode, where uncontrolled surface variability can propagate uncertainty across otherwise well‐controlled experimental designs.

To address interfacial instability, numerous lithium pretreatment strategies have been reported, including chemical etching, mechanical polishing or scraping, and solution‐based formation of artificial solid electrolyte interphases [12, 13, 14, 15, 16, 17, 18, 19, 20, 21, 22]. While these approaches have demonstrated varying degrees of effectiveness, they are typically applied as isolated or empirical procedures, often with disparate parameters and sequences. Consequently, it remains difficult to decouple the individual roles of surface cleaning, morphological homogenization, and chemical interphase formation. More importantly, the absence of a standardized pretreatment framework limits reproducibility and hinders systematic evaluation of lithium surface regulation strategies, thereby also complicating the interpretation of electrochemical results in studies where lithium metal is used as a counter or reference electrode.

Herein, we propose a modular standard operating procedure (SOP) that reformulates lithium metal pretreatment as a sequence of well‐defined and functionally distinct surface‐conditioning steps. The SOP consists of chemical etching (E) to reset the surface chemistry by removing native layers, mechanical brushing (B) to homogenize surface texture and regulate geometry, and solution soaking (S) to induce a controlled artificial interphase through liquid‐phase reactions. By treating these procedures as modular building blocks, different combinations can be constructed and evaluated in a systematic manner, enabling direct comparison of their individual and synergistic contributions to interfacial stability.

Using symmetric Li||Li cells as a diagnostic platform, we first establish the effectiveness of the integrated E–B–S sequence in stabilizing lithium plating and stripping behavior. The symmetric‐cell configuration was selected because it enables direct evaluation of lithium surface and interfacial evolution without additional complications arising from foreign substrates or cathode‐side limitations. Structural and interfacial evolution are then examined through scanning electron microscopy (SEM), lithium‐sensitive energy‐dispersive X‐ray (EDX), atomic force microscopy (AFM), and electrochemical impedance spectroscopy (EIS) to elucidate how the modular SOP governs surface geometry and resistance growth. Density functional theory (DFT) calculations and depth‐resolved X‐ray photoelectron spectroscopy (XPS) are further employed to clarify the chemical origin of controlled etching and artificial interphase formation. Finally, the practical relevance of the standardized lithium surface is validated in Li||LiFePO_4_ full cells, demonstrating that interfacial improvements achieved at the symmetric‐cell level can be translated into practical lithium‐based electrochemical configurations.

## 2 Experimental Section

### 2.1 Lithium Metal Pretreatment and Modular SOP Procedures

Lithium metal foils with an original thickness of approximately 450 µm and a diameter of 15 mm were used as received unless otherwise specified. Except for intentional oxidation, all pretreatment procedures were conducted in an argon‐filled glove box with O_2_ and H_2_O levels below 0.5 ppm. Scheme S1 summarizes the modular roles of oxidation (O), etching (E), brushing (B), and soaking (S) used to construct standardized pretreatment SOPs.

For intentional oxidation, lithium foils were exposed to ambient air for 30 s and then transferred back into the glove box for subsequent processing. Wet chemical etching was carried out using polycyclic aromatic hydrocarbons (PAHs), including biphenyl, naphthalene, and pyrene. Each PAH was dissolved at 3.0 M in tetrahydrofuran (THF) unless otherwise specified. Lithium foils were immersed in the etching solution for 10 min at room temperature, followed by rinsing with fresh THF. The samples were then rested in the glove box for 10 min to ensure complete solvent evaporation. Dry brushing was performed using a nylon soft brush to mechanically homogenize the lithium surface. Brushing was applied uniformly across the lithium surface immediately after the etching step. For soaking within lithium salt solutions, lithium foils were immersed in 1,2‐dimethoxyethane (DME)‐based solutions of LiFSI, LiTFSI, or LiNO_3_ for 5 min, followed by rinsing with DME and resting for 10 min to evaporate residual solvent. For reduced‐lithium‐inventory evaluation using thin lithium foils, lithium metal was mechanically calendered between separator sheets to obtain a final lithium thickness of approximately 50 µm. To minimize excessive lithium consumption and mechanical damage during pretreatment, the etching duration was reduced to 1 min with gentle manual stirring, followed by mild brushing supported by a glass slide during the SOP process. All pretreatment parameters were systematically optimized based on symmetric‐cell performance and reproducibility, and the selected conditions represent the optimal parameter set identified in this study.

### 2.2 Cell Assembly and Electrochemical Measurements

CR2032‐type coin cells were assembled in an argon‐filled glove box (O_2_, H_2_O < 0.5 ppm). For symmetric cells, lithium foils with identical pretreatment histories were used as both working and counter electrodes. For full cells, LiFePO_4_ (LFP) cathodes were paired with lithium metal anodes subjected to different pretreatment procedures. The LFP cathode slurry was prepared using LiFePO_4_, Super P conductive carbon, and polyvinylidene fluoride (PVDF) binder in a weight ratio of 90:4:6, with N‐methyl‐2‐pyrrolidone (NMP) as the solvent. A small amount (1 wt.%) of single‐walled carbon nanotubes was added to improve electronic conductivity. The slurry was cast onto aluminum foil using a doctor blade, dried at 80°C for 1 h, followed by vacuum drying at 120°C for 24 h. Cathode disks with a diameter of 12 mm were punched for cell assembly. A polypropylene separator (Celgard, 20 mm diameter) and an electrolyte consisting of 1.0 M LiPF_6_ in ethylene carbonate/ethyl methyl carbonate (EC/EMC, 1:2 v/v) were used.

Symmetric Li||Li cells were cycled galvanostatically at a constant current of 2 mA cm^−2^ with a plating and stripping duration of 30 min per half‐cycle. Li||Cu asymmetric cells were tested under the same current density and cycling duration, corresponding to an areal capacity of 1 mAh cm^−2^. Li||LFP full cells were initially activated at 0.1 C for three cycles and subsequently cycled at 0.5 C within a voltage window of 2.0–4.0 V. Rate capability was evaluated by stepwise cycling at 0.1, 0.2, 0.3, 0.5, 1.0, and 2.0 C, followed by recovery to 0.1 C. For Li||S full‐cell demonstrations, sulfur catholyte cells were assembled using the Li–biphenyl–sulfur catholyte system reported in our previous work [23]. Cell preparation and testing procedures followed the reported methodology. Electrochemical measurements were conducted using a battery cycler (Lanhe CT3002A). EIS was performed over a frequency range of 1 MHz to 10 mHz with an AC amplitude of 10 mV (BioLogic SP‐50e). Impedance spectra were collected after cell assembly and at selected cycling intervals using the same potentiostat, and fitted using EC‐Lab software.

### 2.3 Structural and Surface Characterizations

Surface and cross‐sectional morphologies of lithium electrodes were examined using field‐emission SEM (FE‐SEM, HITACHI SU‐8010 and SU‐7000). Elemental mapping was performed using a lithium‐sensitive, windowless EDX spectroscopy detector operated at 5 kV. AFM measurements (Bruker, Dimension Icon) were conducted in an argon‐filled glove box to characterize the surface topography of lithium metal electrodes in tapping mode prior to cell assembly. The arithmetic mean roughness (R_a_) represents the average deviation of the surface height from the mean plane, while the root‐mean‐square roughness (R_q_) corresponds to the standard deviation of the height distribution. The surface area difference (SAD) is defined as the percentage increase of the three‐dimensional surface area relative to its projected two‐dimensional area and is used to quantify the effective geometric surface area arising from surface corrugation. XPS measurements (ULVAC PHI, Quantera II) were conducted using a monochromated Al Kα source. Depth profiling was performed using 1 kV Ar^+^ ion sputtering at an estimated etching rate of approximately 12.8 nm min^−1^. All binding energies were calibrated using the C 1s peak at 284.5 eV as a reference.

### 2.4 Density Functional Theory Calculations

All DFT calculations were carried out using the Gaussian 16 software package [24]. The ωB97XD functional in conjunction with the 6‐311+G(d,p) basis set was employed to investigate the interactions between lithium atoms and PAH molecules. Solvation effects were treated using a mixed implicit–explicit approach, in which THF molecules were explicitly included and the bulk solvent effects were modeled using the Integral Equation Formalism Polarizable Continuum Model (IEFPCM) with the dielectric constant of THF (ε = 7.43). Open‐shell species were treated using the unrestricted formalism, and tight self‐consistent field (SCF) convergence criteria were applied throughout. The binding energy (*E_b_*, in eV) was defined as:

$$E_{b}=E_{tot}\left(complex\right)-\sum_{i}E_{i}\left(fragments\right)$$

where *E_tot_* is the total energy of the optimized complex, and $\sum_{i}E_{i}$ represents the sum of the energies of the corresponding isolated fragments with matched stoichiometry, all computed at the same level of theory and using the same solvation model.

## 3 Results and Discussion

### 3.1 Establishing a Modular SOP Framework for Lithium Electrodes

The electrochemical behavior of lithium metal electrodes is highly sensitive to their initial surface state, which is strongly influenced by storage history, surface contamination, and handling variability [19, 25, 26]. Such uncontrolled surface heterogeneity leads to large performance scatter and hampers meaningful comparison across different studies, highlighting the necessity of a standardized, well‐defined pre‐assembly treatment protocol. Rather than treating lithium pretreatment as a single empirical step, a modular framework that allows systematic assessment of individual surface‐conditioning procedures is required. In this work, the lithium metal pretreatment process is decomposed into three modular steps with distinct physical and chemical functions: etching (E), brushing (B), and soaking (S). By recombining these modules into different procedural sequences, their individual and collective contributions to interfacial stability can be directly evaluated without introducing additional variables. Figure 1 presents a systematic comparison of symmetric Li||Li cells assembled with lithium electrodes subjected to different SOP combinations, establishing a modular analysis framework for lithium surface regulation.

**FIGURE 1:**
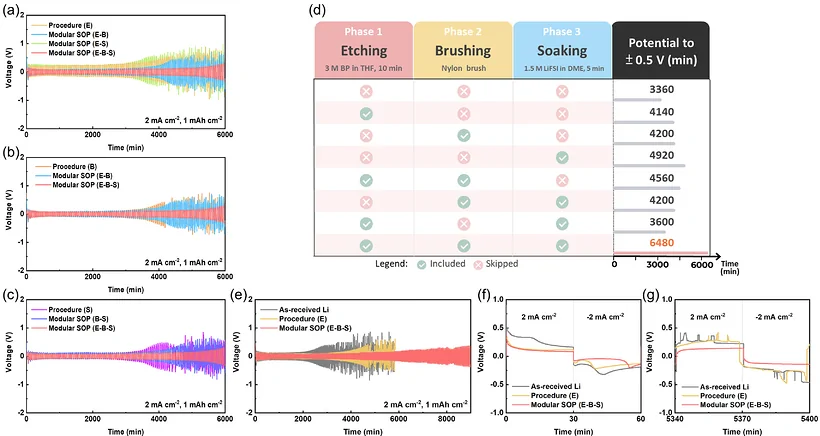
Modular contribution analysis of SOP components in symmetric lithium cells. Voltage profiles of symmetric Li||Li cells comparing (a) etching‐related combinations (E, E–B, E–S, and E–B–S), (b) brushing‐related combinations (B, E–B, and E–B–S), and (c) soaking‐related combinations (S, B–S, and E–B–S). The full E–B–S sequence is included in each panel as a reference. (d) Summary of the time required for the cell voltage to reach ±0.5 V during symmetric cycling. Shorter times indicate earlier voltage divergence, reflecting more rapid interfacial degradation during cycling. (e) Representative voltage profiles of symmetric Li||Li cells using As‐received Li, Procedure (E), and Modular SOP (E–B–S). (f, g) Enlarged view of the selected cycling region showing lithium plating/stripping behavior.

As shown in Figure 1a–c, three groups of SOP combinations are compared by centering on the presence of etching, brushing, and soaking, respectively. In each panel, the complete E–B–S sequence is included as a common reference. Electrodes treated with single‐step or partial SOP combinations generally exhibit earlier voltage amplification and larger fluctuations, indicating rapid interfacial degradation. On the contrary, the fully integrated E–B–S procedure consistently maintains more stable and reproducible voltage profiles over extended cycling. To quantitatively assess the impact of SOP modularity, Figure 1d summarizes the time required for the cell voltage to reach ±0.5 V during symmetric cycling. This metric serves as a practical indicator of interfacial instability and accelerated degradation. SOP combinations lacking one or more key modules show substantially shorter divergence times, reflecting insufficient surface regulation. Notably, the E–B–S sequence significantly delays voltage divergence, suggesting a synergistic effect among the individual modules in stabilizing the lithium interface. These results further indicate that the effects of the individual SOP modules are not simply additive. Direct soaking after etching may induce non‐uniform interphase formation on a freshly exposed and locally reactive lithium surface, whereas the intermediate brushing step helps homogenize the etched surface prior to artificial interphase construction, thereby enabling more continuous interfacial coverage and improved electrochemical stability.

Figure 1e–g further compare representative plating/stripping behaviors of symmetric cells assembled with as‐received lithium, lithium treated by etching alone (Procedure E), and lithium treated by the full modular SOP (E–B–S). Relative to as‐received lithium, Procedure E already leads to a noticeable improvement in voltage stability and smoother cycling behavior, indicating that etching effectively mitigates part of the surface heterogeneity. However, the E–B–S‐treated lithium exhibits the most stable and reproducible voltage profiles, with reduced polarization and minimal fluctuation over repeated cycles. These results demonstrate that while etching provides a meaningful baseline improvement, the additional brushing and soaking steps are necessary to further homogenize the surface state and establish a robust and standardized lithium interface suitable for subsequent structural and chemical analysis.

Additional validation was carried out using Li||Cu asymmetric cells to evaluate lithium plating/stripping reversibility under different SOP treatments (Figures S1 and S2). Compared with as‐received lithium, the E and E–B treatments exhibit partially improved Coulombic efficiency and reduced polarization growth during cycling. Nevertheless, both electrodes gradually develop unstable stripping behavior during extended cycling. In contrast, the E–B–S‐treated lithium maintains a Coulombic efficiency close to 100% with stable plating/stripping voltage profiles over prolonged operation, indicating substantially improved reversibility and interfacial stability on the Cu substrate. These results further support the importance of coupling surface homogenization with controlled artificial interphase formation in the modular SOP framework.

To further examine the applicability of the established SOP, its effectiveness was evaluated on intentionally oxidized lithium surfaces. As shown in Figure S3, symmetric cells assembled with oxidized lithium exhibit significantly elevated overpotential and pronounced voltage fluctuations during cycling. After applying the full modular SOP (O–E–B–S), the cycling behavior is substantially stabilized, with reduced polarization and more symmetric voltage profiles. This robustness against surface degradation further supports the use of the SOP as a unified pretreatment protocol for lithium metal electrodes with diverse surface histories.

### 3.2 Structural and Interfacial Regulation by the Modular SOP

Having established that the modular SOP enables stable and reproducible electrochemical behavior, the next critical question concerns how different pretreatment procedures regulate the surface and interfacial structure of lithium metal electrodes before and after prolonged cycling. To address this issue, both top‐view and cross‐sectional morphologies were systematically examined to elucidate the structural evolution induced by different SOP conditions. The surface morphologies of lithium electrodes prior to cycling are shown in Figure S4a,c,e. As‐received lithium exhibits a highly heterogeneous surface, characterized by pronounced rolling‐induced striations, localized oxidation features, and surface contaminants, reflecting substantial variability originating from manufacturing and storage. After etching treatment (Procedure E), the lithium surface becomes noticeably smoother, indicating that the etching step effectively removes part of the original surface heterogeneity. Following the complete modular SOP (E–B–S), the lithium surface displays a more continuous and uniformly covered morphology, suggesting that the combined procedures further redefine and homogenize the initial surface state.

After extended cycling, the surface differences among the three conditions become more pronounced (Figure S4b,d,f). The as‐received lithium electrode develops a rough and highly nonuniform surface with irregular protrusions and dendritic‐like features, indicative of severe surface degradation induced by repeated lithium plating and stripping [8, 9, 27]. The etched lithium electrode shows minimal improvement in surface uniformity, suggesting that etching alone is insufficient to fully suppress surface deterioration during cycling. In contrast, lithium treated with the full E–B–S SOP retains a relatively flat and continuous surface morphology even after prolonged cycling, demonstrating the effectiveness of the modular SOP in stabilizing the surface structure.

To correlate these surface observations with bulk and interfacial structural evolution, cross‐sectional SEM images and corresponding EDX elemental maps after extended cycling are presented in Figure 2. The as‐received lithium electrode (Figure 2a) exhibits a highly irregular cross‐sectional structure, with a loose and discontinuous interfacial region accompanied by extensive cracks and voids, indicating pronounced structural degradation during cycling. The etched lithium electrode (Figure 2b) also displays pronounced structural damage, characterized by large and continuous cracks extending across the cross‐section. These observations indicate that although etching alters the initial surface chemistry, it is insufficient to suppress bulk cracking or stabilize the structural evolution of lithium during prolonged cycling. In contrast, lithium electrodes treated with the full modular SOP (E–B–S) exhibit the most uniform and continuous cross‐sectional structure after cycling (Figure 2c). The interfacial region appears denser, with significantly reduced cracking and porosity. Importantly, the use of a lithium‐sensitive EDX detector enables direct visualization of lithium redistribution across the cross‐section, allowing the redeposited lithium layer to be clearly distinguished from the underlying bulk lithium metal. The corresponding EDX maps reveal that the distributions of Li, F, P, O, C, and S are more continuous and spatially uniform for the E–B–S‐treated electrode compared with the as‐received and etched counterparts, indicating a lower degree of interfacial compositional heterogeneity.

**FIGURE 2:**
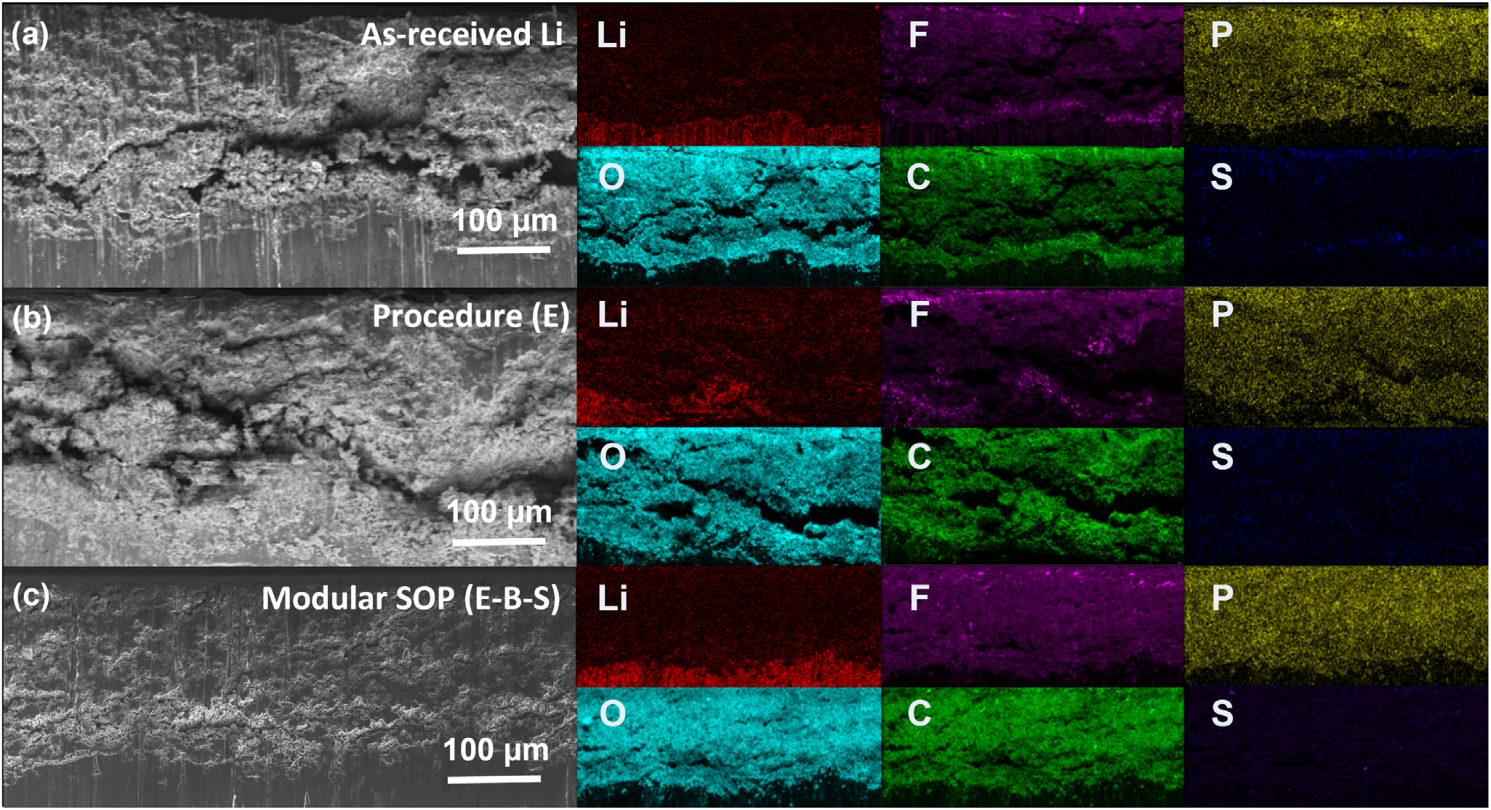
SEM cross‐sectional images of lithium electrodes prepared using (a) As‐received Li, (b) Procedure (E), and (c) Modular SOP (E–B–S) after extended cycling, together with corresponding EDX elemental maps. Elemental distributions of Li, F, P, O, C, and S were collected using a dedicated lithium‐sensitive detector.

Distinct differences in the spatial distribution of fluorine are observed among the different pretreatment conditions. For the as‐received lithium and etched lithium electrode, the F signal appears sparse and unevenly distributed across the interfacial region. In contrast, the E–B–S‐treated lithium electrode exhibits a more continuous and homogeneous fluorine distribution along the interface. This observation suggests that the modular SOP promotes the formation of a chemically more uniform interfacial region, providing important clues for the subsequent chemical analysis of fluorine‐containing species discussed later. As a result, the combined surface observations before and after cycling and the cross‐sectional and elemental analyses after cycling demonstrate that the modular SOP effectively regulates the initial surface state of lithium metal and, in turn, influences its structural evolution and interfacial uniformity during prolonged cycling.

AFM was employed to evaluate how the modular SOP regulates the surface geometry of lithium metal treated with different pretreatment sequences (Figure 3 and Figure S5). As‐received lithium exhibits pronounced height fluctuations and directionally aligned surface features characteristic of the rolling process used during lithium foil fabrication. The corresponding surface roughness parameters show a root‐mean‐square roughness (R_q_) of approximately 61 nm and an arithmetic mean roughness (R_a_) of 51 nm, while the surface area difference (SAD) remains low at ∼1.1%, indicating that the geometric surface area is only marginally larger than the projected area despite the presence of rolling‐induced textures. After etching treatment (Procedure E), the lithium surface becomes visibly smoother, accompanied by a narrowed height distribution and substantial attenuation of the rolling‐induced features. The roughness parameters decrease to approximately 30 nm (R_q_) and 23 nm (R_a_). Meanwhile, the SAD increases only slightly to ∼1.9%, suggesting that the primary effect of etching is surface leveling rather than the creation of additional geometric complexity.

**FIGURE 3:**
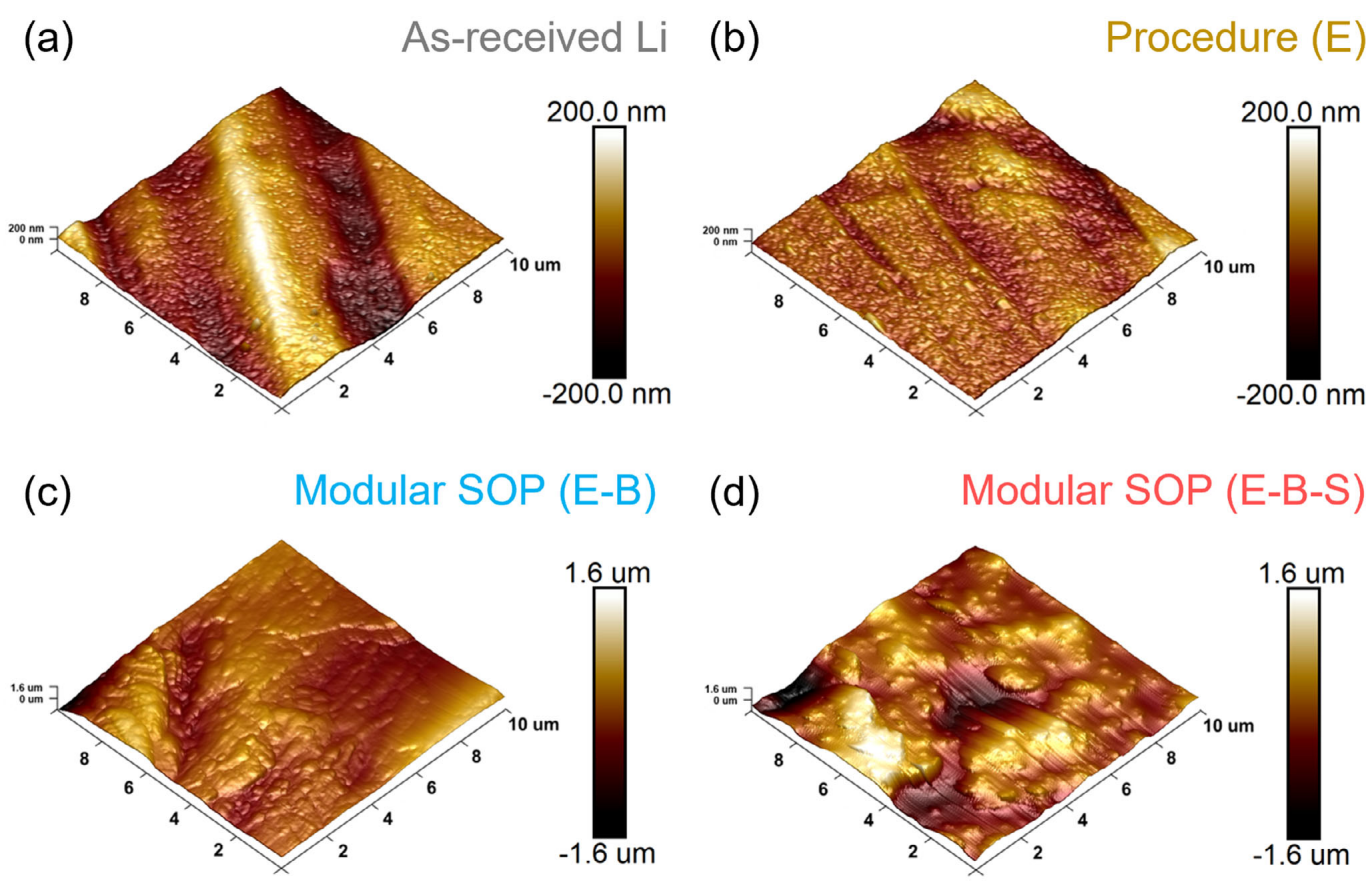
AFM characterization of lithium surface evolution induced by different pretreatment procedures. AFM height images of lithium surfaces prepared using (a) As‐received Li, (b) Procedure (E), (c) Modular SOP (E–B), and (d) Modular SOP (E–B–S).

A markedly different surface morphology emerges after the combined etching and brushing sequence (E–B). The AFM images reveal a transition from directionally aligned features to a three‐dimensionally corrugated surface with large height variations. This change is reflected quantitatively by increases in R_q_ and R_a_ to approximately 253 and 195 nm, respectively, together with a pronounced rise in SAD to ∼18.8%. These results indicate that brushing plays a central role in introducing surface corrugation and expanding the effective geometric surface area. Following the complete modular SOP (E–B–S), the lithium surface displays the highest degree of geometric complexity among the examined conditions. The roughness parameters further increase, reaching approximately 430 nm (R_q_) and 316 nm (R_a_), while the SAD rises to ∼44.7%. The preservation of a high SAD after the soaking step indicates that the surface geometry introduced by brushing remains structurally intact, yielding a continuous three‐dimensional surface topology rather than isolated asperities. This suggests that the subsequently formed artificial interphase conforms to, and integrates with, the three‐dimensionally structured lithium surface rather than flattening or passivating it. The AFM results clarify the distinct roles of the individual SOP modules in shaping the surface geometry of lithium metal. Etching reduces native surface heterogeneity and establishes a uniform baseline, brushing substantially increases the effective surface area through geometric corrugation, and the full E–B–S sequence preserves and further amplifies this high‐area morphology. From a geometric perspective, an expanded effective surface area corresponds to a lower local current density under identical applied currents [28, 29], providing a structurally favorable starting point for subsequent interfacial evolution.

EIS was employed to examine how the modular SOP influences the interfacial resistance evolution of lithium metal during cycling, as shown in Figure 4. The impedance spectra were analyzed using an equivalent circuit model, where the high‐frequency intercept corresponds to the electrolyte resistance (R_e_), while the first and second semicircles are assigned to the interphase‐related resistance (R_SEI_) and the charge‐transfer resistance (R_ct_), respectively [16]. As shown in Figure 4a, prior to cycling, the as‐received lithium electrode exhibits a relatively large R_SEI_, indicating an unfavorable initial interfacial state. In contrast, lithium treated with the complete modular SOP (E–B–S) displays a substantially lower initial R_SEI_, suggesting that the pretreatment effectively modifies the interfacial characteristics of lithium metal. After 50 h of cycling (Figure 4b), both R_SEI_ and R_ct_ of the as‐received lithium electrode decrease temporarily, indicating an initial interfacial activation and reorganization process during early cycling. With further cycling to 100 h (Figure 4c), the impedance semicircles expand markedly, accompanied by concurrent increases in R_SEI_ and R_ct_. This trend reflects progressive interfacial degradation under prolonged cycling, where resistance accumulation becomes dominant.

**FIGURE 4:**
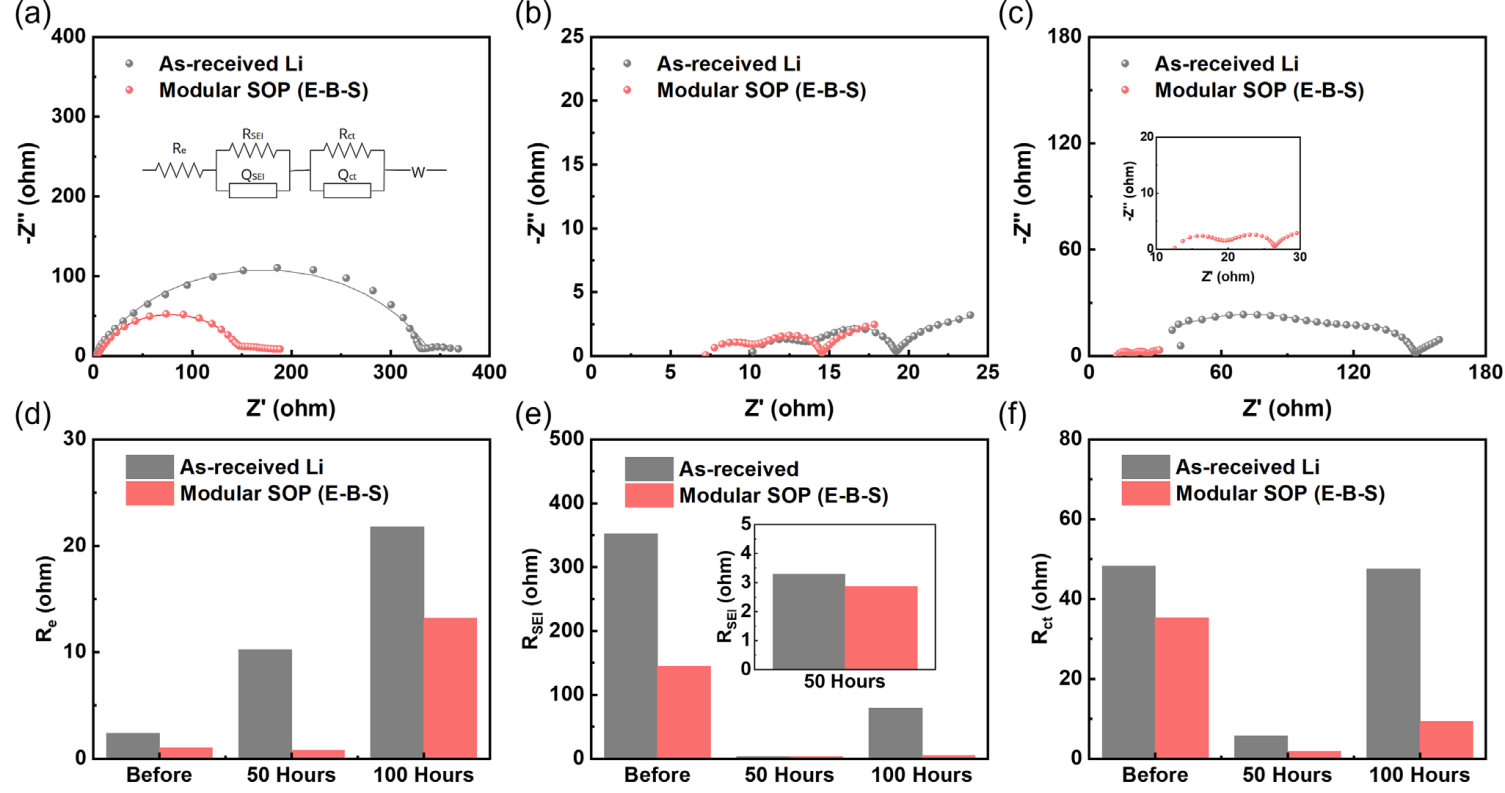
Interfacial resistance evolution revealed by EIS. Nyquist plots of symmetric Li||Li cells assembled with As‐received Li and Modular SOP (E–B–S) (a) before cycling and after (b) 50 and (c) 100 h of cycling. Comparison of interfacial resistance components extracted from equivalent circuit fitting, including (d) electrolyte resistance (R_e_), (e) SEI resistance (R_SEI_), and (f) charge‐transfer resistance (R_ct_).

In comparison, lithium electrodes treated with the E–B–S SOP exhibit a distinctly different impedance evolution. Both R_SEI_ and R_ct_ remain relatively stable after 50 h of cycling, and their increases after 100 h are significantly smaller than those observed for as‐received lithium. These results demonstrate that the modular SOP effectively suppresses the long‐term growth of interfacial resistance during extended cycling. The fitted impedance parameters summarized in Figure 4d–f further highlight these differences. While R_e_ varies with cycling time for both electrodes, lithium treated with the E–B–S SOP consistently maintains a lower R_e_ across all measured states. This behavior suggests a more stable electrolyte–interface environment and implies that the stabilized lithium interface is less prone to inducing electrolyte degradation during prolonged operation. In combination with the SEM observations of a dense and continuous interfacial structure and the AFM results revealing a high‐surface‐area yet geometrically uniform initial lithium surface, the EIS analysis indicates that lithium electrodes processed by the E–B–S modular SOP possess interfacial architectures that are more effective in mitigating resistance accumulation over long‐term cycling.

### 3.3 Chemical Origin of Controlled Surface Regulation by the Etching–Soaking Module

To examine how the choice of PAHs influences the etching behavior of lithium metal under the E–B–S modular SOP, pyrene, naphthalene, and biphenyl were employed as etching agents in THF [30]. The electrochemical responses of symmetric Li||Li cells after etching are compared in Figure 5a. Lithium etched with pyrene exhibits pronounced voltage fluctuations during early cycling and fails within approximately 2800–3000 min, indicating an unstable interfacial state. Naphthalene‐treated lithium shows moderately improved stability, sustaining cycling for around 3800–4000 min, but still displays noticeable polarization growth. In comparison, biphenyl‐treated lithium delivers the most stable voltage profiles and the longest cycling lifetime, extending to over 6000 min with suppressed overpotential evolution. Consistent trends are observed in the etching‐rate comparison shown in Figure 5b. Pyrene induces the fastest lithium mass loss, followed by naphthalene and biphenyl. The slower and more controlled etching behavior of biphenyl suggests a more uniform surface modification process, which is beneficial for subsequent interfacial stabilization.

**FIGURE 5:**
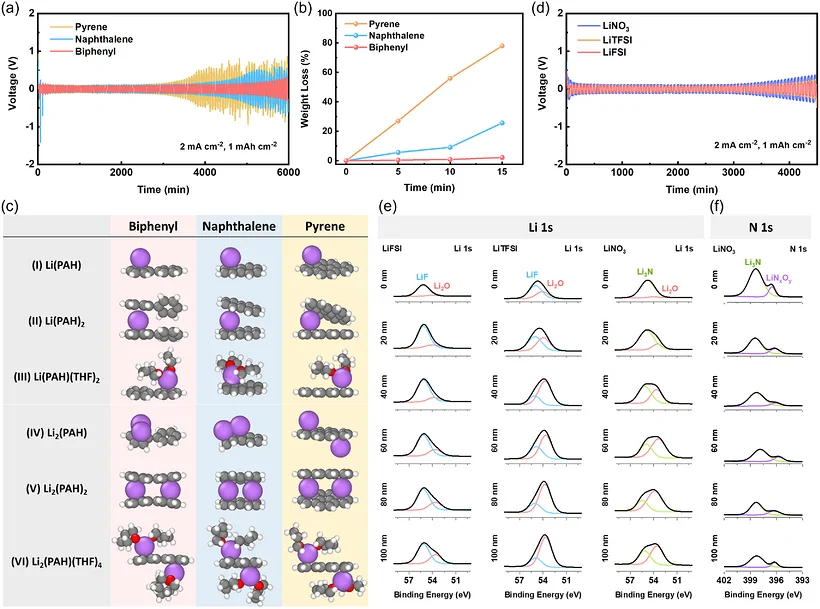
Chemical regulation of lithium surfaces through PAH‐assisted etching and lithium‐salt soaking. (a) Voltage profiles of symmetric Li||Li cells using three different PAHs in THF as etching solutions, followed by brushing and soaking in 1.0 M LiFSI in DME. (b) Mass loss of lithium foil as a function of etching time in THF solutions containing different PAHs. (c) DFT‐optimized coordination structures of lithium–PAH systems, including single‐lithium configurations: (I) Li(PAH), (II) Li(PAH)_2_, and (III) Li(PAH)(THF)_2_, as well as double‐lithium configurations: (IV) Li_2_(PAH), (V) Li_2_(PAH)_2_, and (VI) Li_2_(PAH)(THF)_4_. Gray, white, purple, and red spheres represent C, H, Li, and O atoms, respectively. (d) Voltage profiles of symmetric Li||Li cells after lithium foil pretreatment by soaking in different lithium salt solutions (0.5 m LiFSI, LiTFSI, and LiNO_3_ in DME). XPS depth profiles of the (e) Li 1s and (f) N 1s spectra for lithium surfaces treated with different lithium salt soaking solutions.

DFT calculations were performed to evaluate the interactions among lithium, PAH molecules, and coordinating THF solvent, as shown in Figure 5c and Figure S6. Both single‐lithium and double‐lithium coordination configurations were considered. The calculated binding energies (*E_b_*) follow the trend pyrene > naphthalene > biphenyl, indicating progressively stronger Li–PAH interactions with increasing *π*‐conjugation. Pyrene therefore exhibits the strongest affinity toward lithium [31, 32], which is consistent with the most rapid mass loss observed in Figure 5b. In contrast, the moderate interaction strength between lithium and biphenyl enables controlled etching; such affinity is sufficient to remove native surface layers while preventing aggressive lithium loss. This balance explains why biphenyl‐assisted etching produces a more homogeneous lithium surface and leads to superior electrochemical stability.

Following biphenyl solution etching and surface homogenization via brushing, lithium foils were further treated by soaking in LiNO_3_, LiTFSI, or LiFSI solutions to induce artificial interphase formation [33, 34, 35, 36, 37, 38]. The corresponding symmetric‐cell performances are compared in Figure 5d. Lithium treated with LiNO_3_ shows earlier voltage divergence during cycling, occurring at approximately 3300–3500 min. Lithium treated with LiTFSI and LiFSI exhibits more stable voltage evolution over extended cycling. Between the two fluorinated salts, LiFSI‐treated lithium shows slightly reduced polarization growth and marginally extended cycling stability, reaching more than 4500 min, although the difference relative to LiTFSI remains limited. These results suggest that fluorinated salts provide more effective interfacial stabilization than nitrate‐based treatments under the present conditions.

XPS depth profiling was conducted to correlate the electrochemical behavior with interfacial chemistry, as shown in Figure 5e,f and Figure S7. Distinct chemical signatures are observed for lithium surfaces treated with different salts. For LiNO_3_‐treated lithium, nitrogen‐containing species dominate the interphase, including Li_3_N (Li 1s ∼54.8–55.4 eV; N 1s ∼397.8–398.4 eV) and LiN_x_O_y_ (N 1s ∼395.7–396.5 eV) [27, 39]. These species persist into deeper regions of the interphase, indicating the formation of a nitrate‐derived inorganic layer. Lithium treated with LiFSI exhibits a pronounced LiF‐rich interphase, as evidenced by strong LiF signals (Li 1s ∼54.9–55.2 eV; F 1s ∼684.5–685.1 eV) extending from the surface into subsurface regions [34, 39, 40]. Li_2_O is also detected (Li 1s ∼53.3–53.9 eV; O 1s ∼527.5–528.4 eV) [27, 34, 39, 41], but its contribution remains secondary. In contrast, LiTFSI‐treated lithium shows LiF formation mainly near the surface, with a rapid decrease in fluorine intensity upon sputtering (Figure S7b). These depth‐resolved chemical profiles indicate that LiFSI promotes the formation of a LiF‐rich artificial interphase that extends into the three‐dimensionally structured lithium surface, resulting in a more continuous interfacial coverage. Given the high chemical stability and mechanical robustness of LiF [36, 40, 42, 43], such a spatially extended LiF distribution is favorable for suppressing parasitic reactions and maintaining interfacial integrity during repeated lithium plating and stripping. This compositional feature is consistent with the reduced polarization growth and improved cycling stability observed in symmetric cells.

In addition to electrochemical stabilization, recent studies have suggested that stable inorganic‐rich interphases and dendrite‐suppressed lithium deposition may help mitigate localized side reactions and interfacial thermal instability in lithium metal batteries [44]. In particular, unstable SEI rupture/regeneration and dendritic lithium accumulation have been closely associated with accelerated thermal runaway behavior in cycled lithium metal systems [45]. Therefore, constructing chemically stable and spatially uniform artificial interphases may also be beneficial for improving interfacial robustness under aggressive operating conditions.

### 3.4 Full‐Cell Validation of the Standardized Lithium Electrodes

The effectiveness of the modular SOP established above was further evaluated in practical Li||LFP full cells to assess whether the interfacial improvements observed in symmetric cells can be translated to realistic battery configurations. The electrochemical performance of full cells assembled with as‐received lithium and SOP‐treated lithium (E–B–S) is summarized in Figure 6. Figure 6a compares the cycling stability of Li||LFP full cells operated at 0.5 C after the formation cycles at 0.1 C. While both cells exhibit comparable initial capacities, their long‐term behaviors differ significantly. The full cell using as‐received lithium suffers from rapid capacity decay, retaining only 39.6% of its initial capacity after 200 cycles. In contrast, the cell assembled with E–B–S‐treated lithium maintains 90.2% capacity retention after the same number of cycles, demonstrating a substantial improvement in cycling stability. This result indicates that interfacial instability of lithium metal, if left unregulated, is readily amplified at the full‐cell level, whereas standardized surface treatment effectively mitigates this degradation pathway.

**FIGURE 6:**
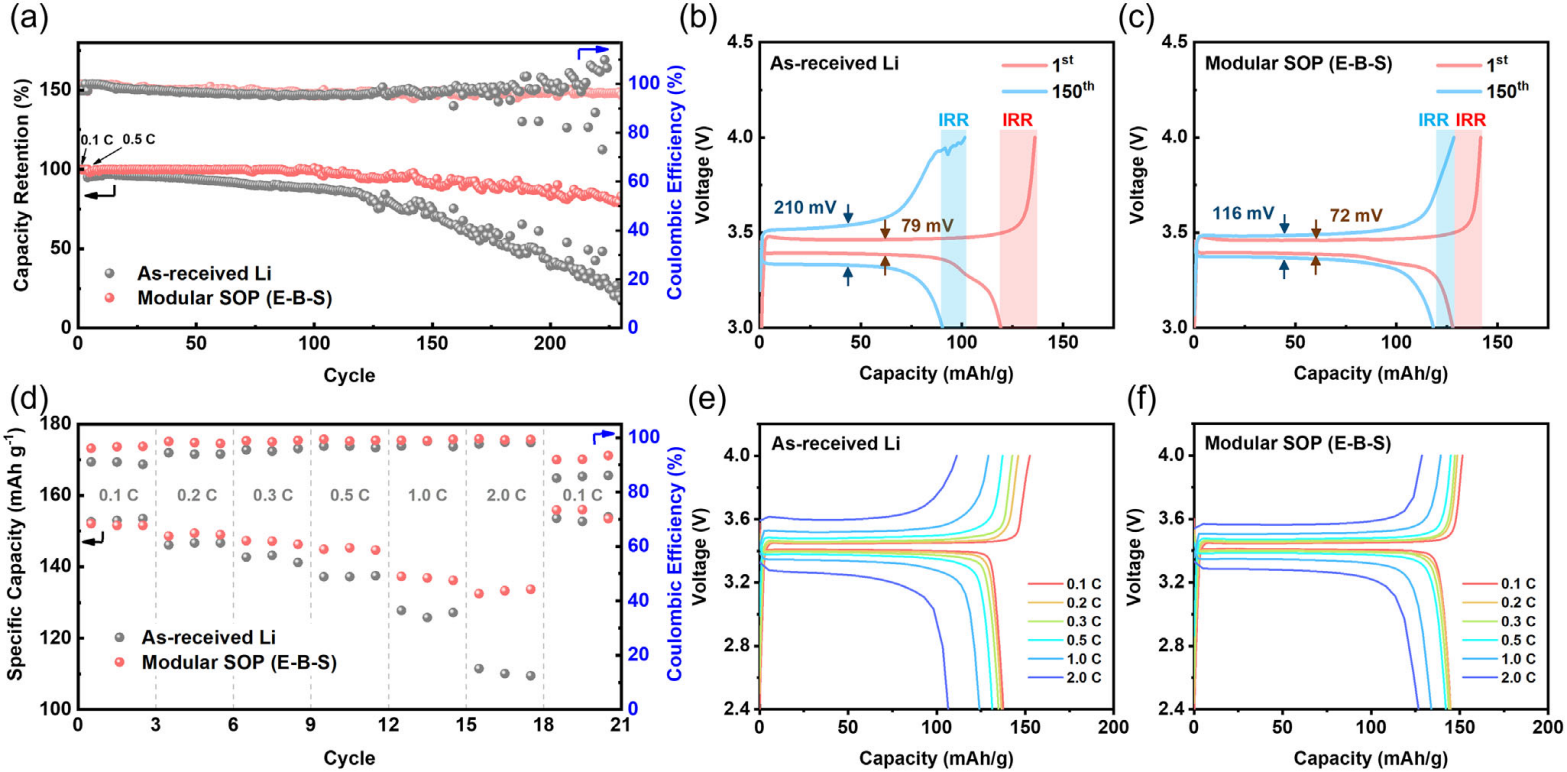
Electrochemical performance of Li||LFP full cells assembled with As‐received Li and Modular SOP (E–B–S). (a) Cycling stability comparison of full cells using different lithium anodes. Representative voltage profiles of full cells using (b) As‐received Li and (c) Modular SOP (E–B–S) at 0.5 C. (d) Rate capability of Li||LFP full cells over a range of current densities. Representative voltage profiles at different rates for full cells using (e) As‐received Li and (f) Modular SOP (E–B–S).

Representative charge–discharge voltage profiles further illustrate the differences in interfacial behavior (Figure 6b,c). At the first cycle, the overpotentials of cells using as‐received lithium and E–B–S‐treated lithium are similar, measuring 79 mV and 72 mV, respectively. Upon extended cycling to the 150th cycle, however, the overpotential of the as‐received lithium cell increases substantially to 210 mV, reflecting pronounced polarization growth. By comparison, the E–B–S‐treated lithium cell exhibits a much smaller increase, reaching 116 mV at the same cycle number. In addition, cells with as‐received lithium display larger irreversible capacity losses (IRR) on a per‐cycle basis and more pronounced voltage instability, indicating poor reversibility of lithium plating and stripping. These effects are significantly alleviated by the modular SOP, consistent with a more stable and uniform lithium interface.

Rate capability measurements further highlight the benefits of the standardized lithium surface (Figure 6d). At elevated current densities, the performance gap between the two cells becomes increasingly apparent. Full cells using as‐received lithium deliver specific capacities of 124 mAh g^−1^ at 1 C and 106 mAh g^−1^ at 2 C (Figure 6e). In contrast, cells assembled with E–B–S‐treated lithium maintain higher capacities of 134 mAh g^−1^ at 1 C and 127 mAh g^−1^ at 2 C (Figure 6f). The corresponding voltage profiles confirm reduced polarization and more stable voltage plateaus across a wide range of current densities when the SOP‐treated lithium anode is employed. To sum up, the full‐cell results demonstrate that the modular SOP developed in this work provides tangible benefits beyond symmetric‐cell testing. By stabilizing the lithium metal interface prior to cell assembly, the E–B–S procedure enables improved capacity retention, suppressed polarization growth, and enhanced rate capability in Li||LFP full cells, highlighting its practical relevance for lithium metal battery applications.

To further assess the applicability of the SOP under reduced lithium inventory conditions, additional Li||LFP full‐cell tests were conducted using 50 µm calendered Li foils (Figure S8). Representative charge–discharge voltage profiles reveal that the untreated lithium electrode exhibits progressively increasing polarization during cycling, as evidenced by the growing separation between charge and discharge plateaus from the third to the 50^th^ cycle. In contrast, the E–B–S‐treated lithium maintains substantially lower polarization and more stable voltage profiles throughout cycling. Furthermore, Li||LFP full cells employing E–B–S‐treated 50 µm calendered Li exhibit stable cycling performance over 100 cycles. These results suggest that the interfacial benefits provided by the SOP remain effective under thin‐lithium conditions. In addition, preliminary Li||S full‐cell tests were performed using E–B–S‐treated thin lithium electrodes (Figure S9). The cells deliver specific capacities of 1192 and 1035 mAh g^−1^ at 0.1 C and 0.2 C, respectively, indicating effective sulfur utilization. Stable charge–discharge behavior was observed at both rates, and reversible cycling could be achieved. These results demonstrate that the SOP‐treated lithium can also be successfully integrated into sulfur‐based battery systems, highlighting the potential applicability of the proposed framework across different cathode chemistries.

Beyond the electrochemical improvements demonstrated above, the modular nature of the proposed SOP also provides potential compatibility with scalable lithium‐metal processing workflows. Since etching, brushing, and soaking are physically separated and independently tunable, the framework may be adaptable to continuous or roll‐to‐roll lithium pretreatment systems. Nevertheless, several practical challenges remain for large‐scale implementation, including etching‐solution aging, solvent recovery, brush‐material wear, and compositional drift of the soaking solution during repeated operation. Therefore, further engineering optimization and inline process control will be necessary to translate the present laboratory‐scale SOP into practical assembly‐line manufacturing environments.

Scheme 1 provides a conceptual overview of the pre‐assembly modular SOP proposed for lithium metal surface standardization. Etching resets the chemical state of the lithium surface by removing native layers, brushing homogenizes surface morphology and mitigates local heterogeneity, and soaking introduces a controlled artificial interphase through liquid‐phase contact. Through systematic evaluation and combination of these modules, the integrated E–B–S sequence is identified as the optimal SOP for achieving a reproducible and stable lithium interface. This scheme emphasizes that the present work establishes not a single surface treatment, but a standardized and transferable protocol that can be readily implemented as a pre‐assembly strategy for stabilizing lithium metal interfaces.

**SCHEME 1:**
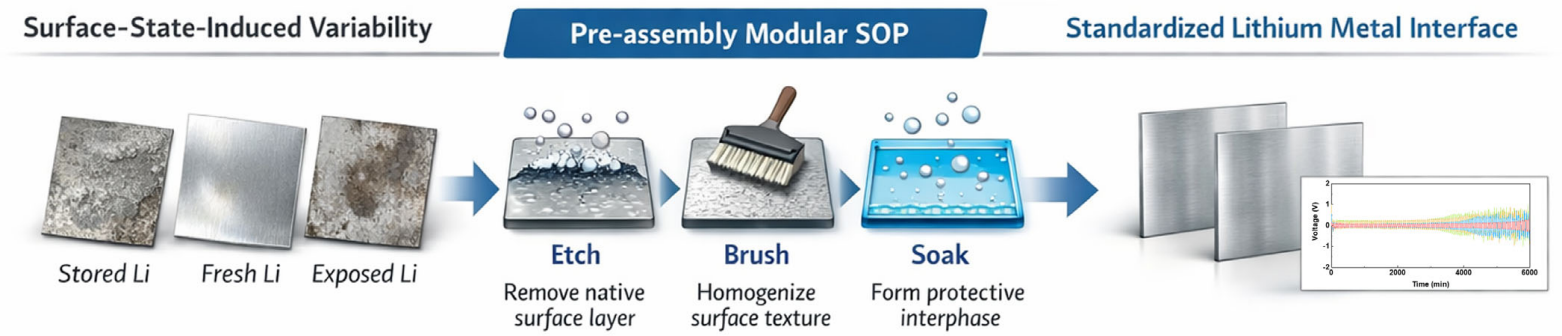
Conceptual illustration of a pre‐assembly modular SOP for standardizing lithium metal surfaces. The scheme summarizes the modular roles of etching, brushing, and soaking and illustrates the identification of the integrated sequence as the optimal SOP for achieving a reproducible and stable lithium interface.

## 4 Conclusions

In this work, we establish a modular and standardized pre‐assembly SOP that enables controlled regulation of lithium metal surfaces prior to cell assembly. By decoupling lithium pretreatment into etching, brushing, and soaking modules, the proposed framework allows systematic evaluation of how physical and chemical surface conditioning governs subsequent interfacial evolution. Rather than relying on empirical or case‐specific treatments, this approach provides a reproducible starting point for investigating lithium metal interfaces.

Through combined electrochemical, structural, and spectroscopic analyses, the modular SOP is shown to simultaneously regulate surface geometry, interfacial chemistry, and resistance evolution during cycling. The resulting lithium interface exhibits improved uniformity, suppressed polarization growth, and enhanced stability in both symmetric cells and practical Li||LFP full cells. These improvements arise not from a single modification step, but from the synergistic integration of surface homogenization and controlled interphase formation.

Beyond performance enhancement, the significance of this work lies in enabling standardization. By minimizing uncontrolled variability associated with lithium surface states, the proposed SOP facilitates meaningful comparison across studies and improves the interpretability of interfacial characterization results. This modular framework is readily adaptable to different electrolytes, cell configurations, and lithium‐handling conditions, and provides a general strategy for stabilizing lithium metal interfaces by transforming lithium metal from an ill‐defined material into a surface‐defined and interface‐stabilized system suitable for rigorous mechanistic investigation rather than empirical optimization.

## Author Contributions

**Han‐Shiuan Lin**: data curation, software, formal analysis. **Anlin Shaju**: data curation, investigation, validation, formal analysis. **Quang Huy Dinh**: formal analysis, data curation. **Sih‐Ling Hsu**: software, visualization, data curation, formal analysis. **Yu‐Sheng Su**: methodology, conceptualization, investigation, validation, supervision, resources, project administration, visualization, writing – review and editing, writing – original draft, funding acquisition, formal analysis. **Che‐Ning Yeh**: supervision, resources, validation, writing – review and editing. **Wen‐Hsin Chang**: investigation, methodology, formal analysis, data curation, writing – original draft, visualization, validation. **Elise Yu‐Tzu Li**: writing – review and editing, validation, supervision, resources, methodology, software.

## Conflicts of Interest

The authors declare no conflicts of interest.

## Supporting information




**Supporting File**: advs76595‐sup‐0001‐SuppMat.docx.

## Data Availability

The data that support the findings of this study are available from the corresponding author upon reasonable request.
