# Supplementary material for: A Modular Standard Operating Procedure for Standardizing Lithium Metal Interfaces
Source: Adv Sci (Weinh). 2026 Jul 15:e76595. Online ahead of print. doi: 10.1002/advs.76595 (PMC13370193; doi:10.1002/advs.76595)
Supplement: Supplementary file 1 — Supporting File: advs76595‐sup‐0001‐SuppMat.docx. [file ADVS-9999-e76595-s001.docx]

**Supporting Information**

**A Modular Standard Operating Procedure for Standardizing Lithium Metal Interfaces**

Wen-Hsin Chang^1^, Anlin Shaju^1^, Han-Shiuan Lin^2^, Sih-Ling Hsu^3^, Quang Huy Dinh^1^, Che-Ning Yeh^2^, Elise Yu-Tzu Li^3^ and Yu-Sheng Su*^1,4^

^1^International College of Semiconductor Technology, National Yang Ming Chiao Tung University, 1001 Daxue Road, Hsinchu 300093, Taiwan

^2^Department of Materials Science and Engineering, National Tsing Hua University, 101, Section 2, Kuang-Fu Road, Hsinchu 300044, Taiwan

^3^Department of Chemistry, National Taiwan Normal University, No. 88, Section 4, Tingzhou Road, Taipei 11677, Taiwan

^4^Industry Academia Innovation School, National Yang Ming Chiao Tung University, 1001 Daxue Road, Hsinchu 300093, Taiwan

*Corresponding author: Yu-Sheng Su ([yushengsu@nycu.edu.tw](mailto:yushengsu@nycu.edu.tw))


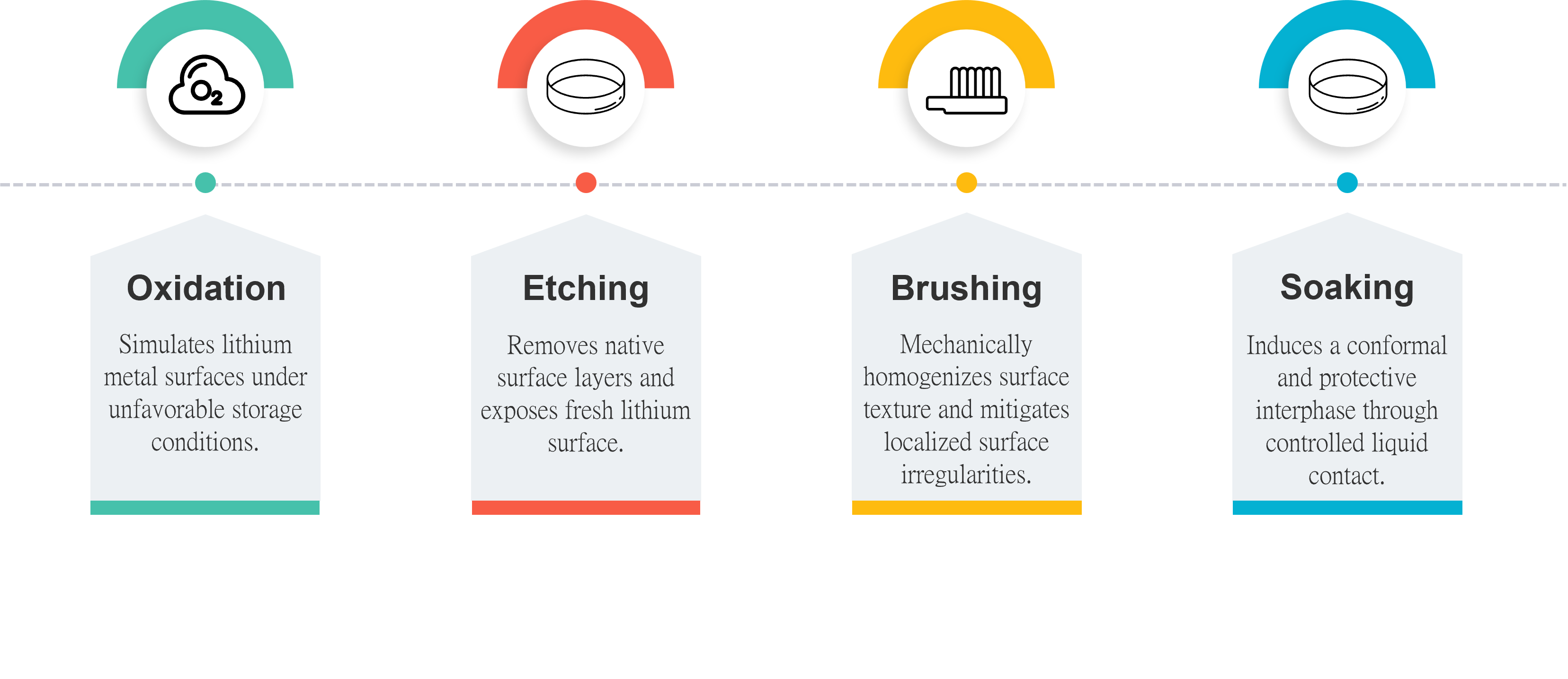


Scheme S1. Functional roles of individual surface procedures in lithium metal pretreatment. Schematic summary illustrating the functional roles of individual procedures involved in lithium metal pretreatment. Oxidation is included as a reference condition to simulate unfavorable storage, while etching (E), brushing (B), and soaking (S) represent modular procedures that collectively contribute to surface homogenization and interfacial stabilization.


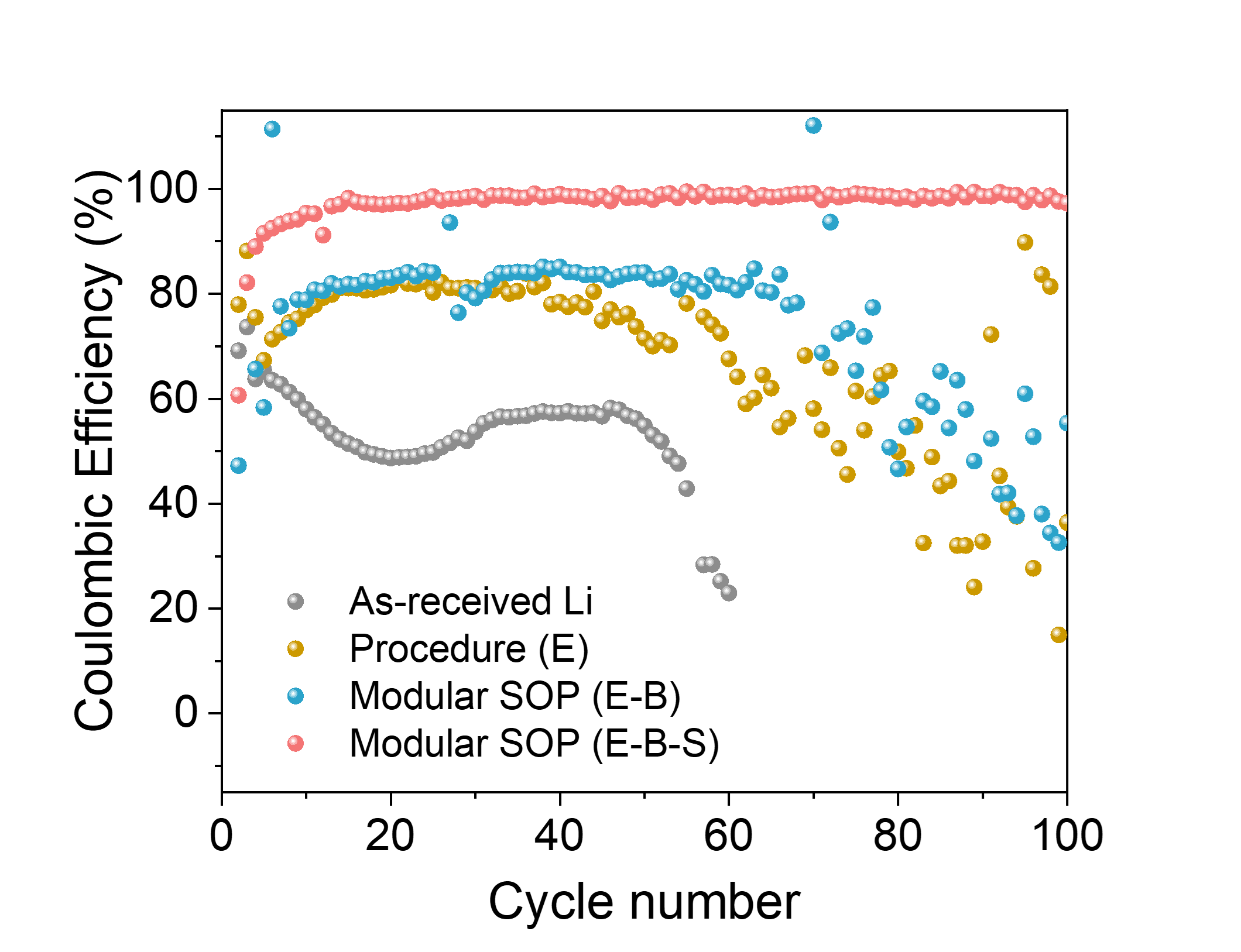


Figure S1. Li||Cu asymmetric-cell evaluation of lithium electrodes subjected to different pretreatment procedures. Coulombic efficiency comparison of Li||Cu cells using As-received Li, Procedure (E), Modular SOP (E–B), and Modular SOP (E–B–S) during cycling.


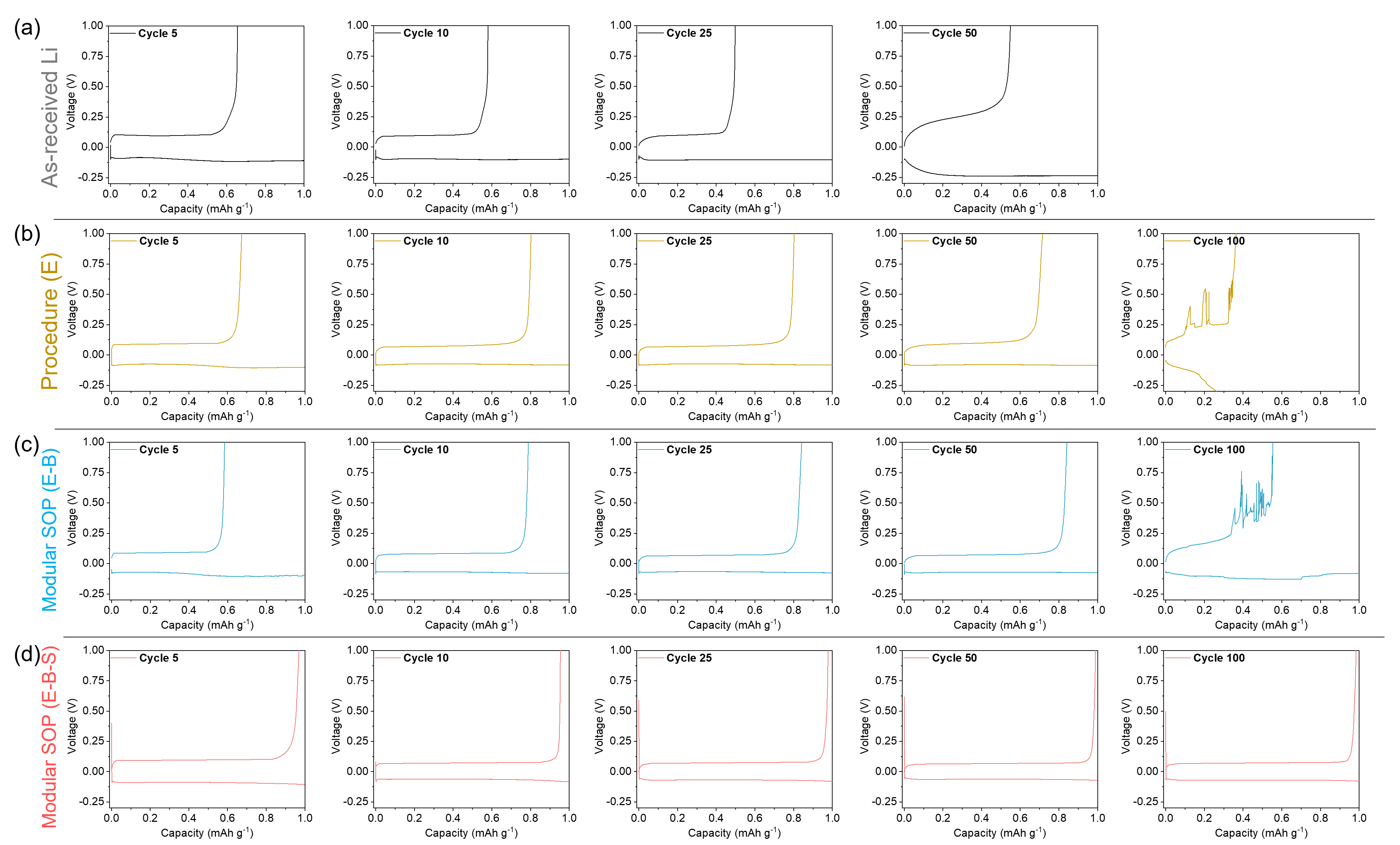


Figure S2. Representative plating/stripping voltage profiles of Li||Cu cells using (a) As-received Li, (b) Procedure (E), (c) Modular SOP (E–B), and (d) Modular SOP (E–B–S).


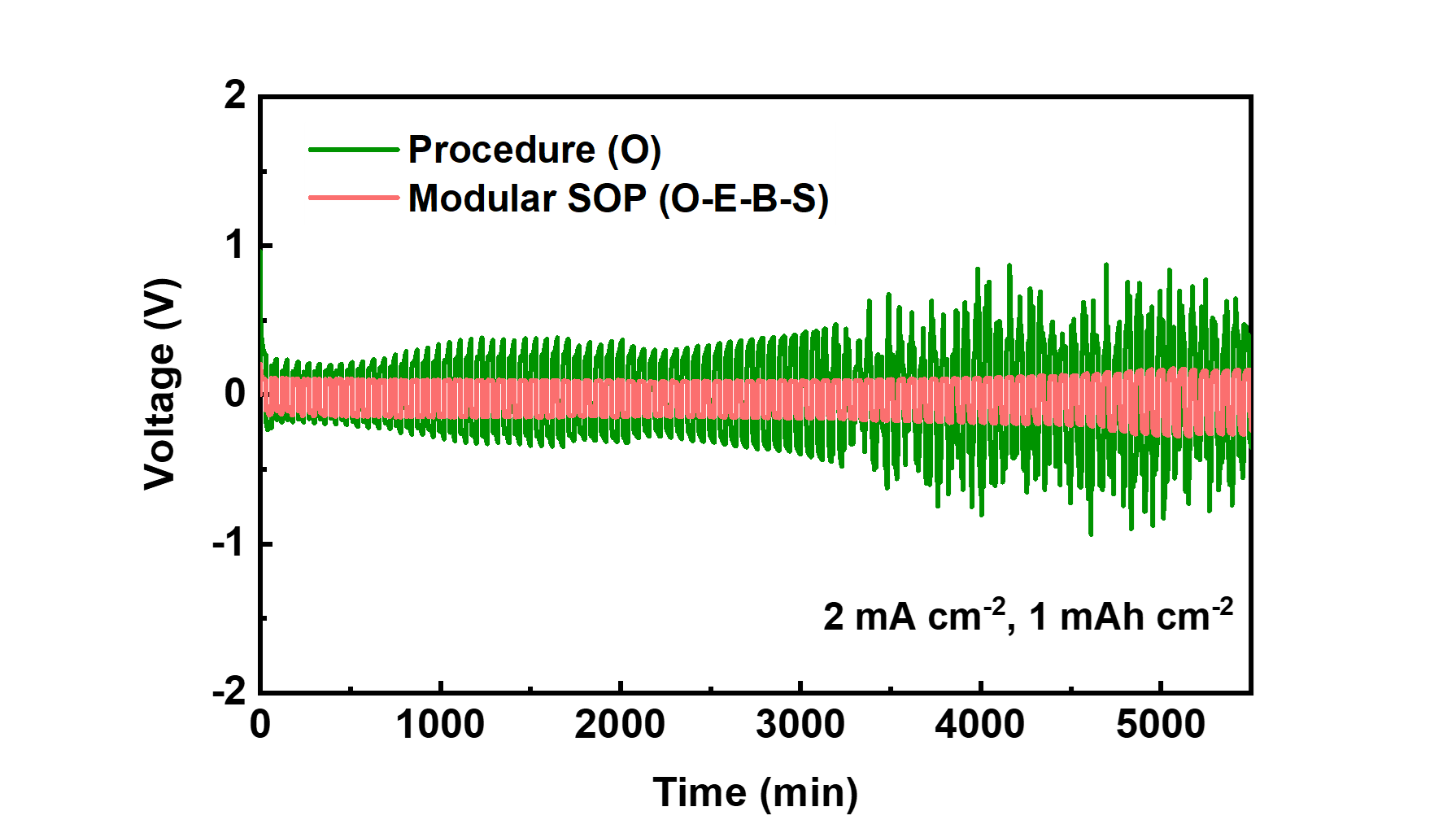


Figure S3. Representative voltage profiles of symmetric Li||Li cells assembled with oxidized lithium foil treated by Procedure (O) and using Modular SOP (O–E–B–S), demonstrating the applicability of the developed SOP to degraded lithium surfaces.


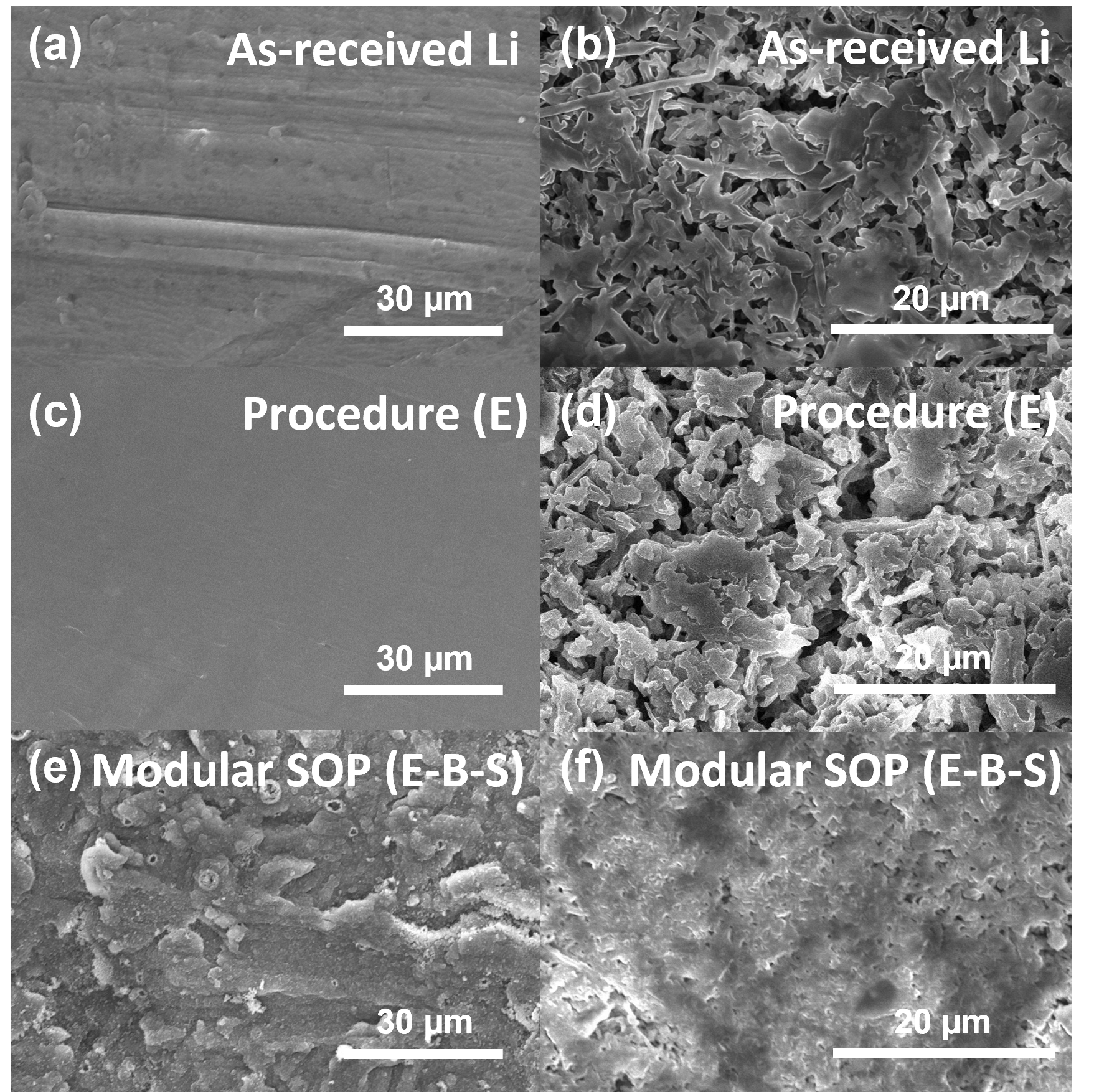


Figure S4. SEM top-view images of lithium electrodes prepared using As-received Li, Procedure (E), and Modular SOP (E–B–S) shown (a, c, e) before cycling and (b, d, f) after extended cycling.


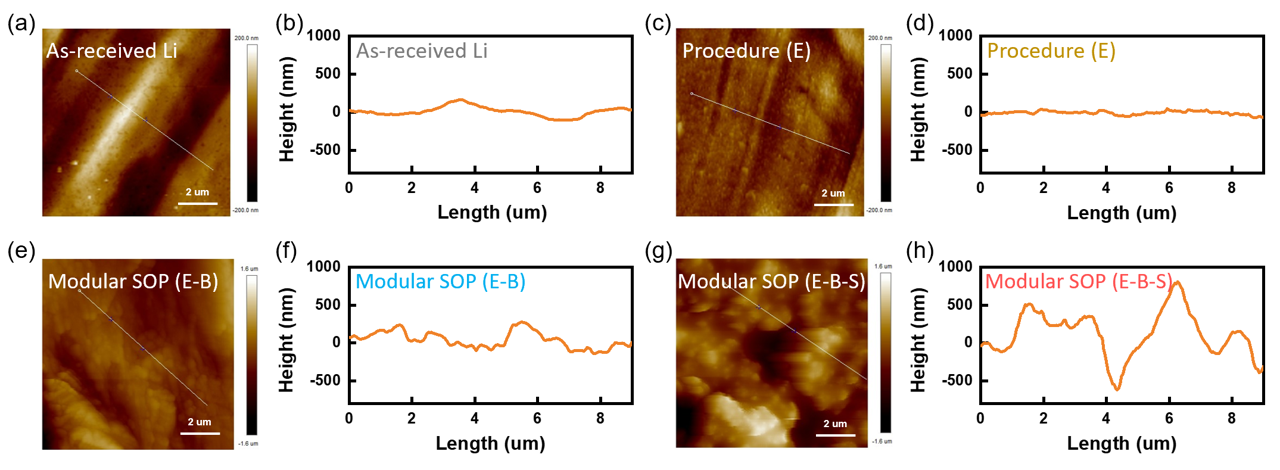


Figure S5. (a, c, e, g) AFM height images and (b, d, f, h) corresponding line profiles of lithium surfaces prepared using As-received Li, Procedure (E), Modular SOP (E–B), and Modular SOP (E–B–S), respectively.


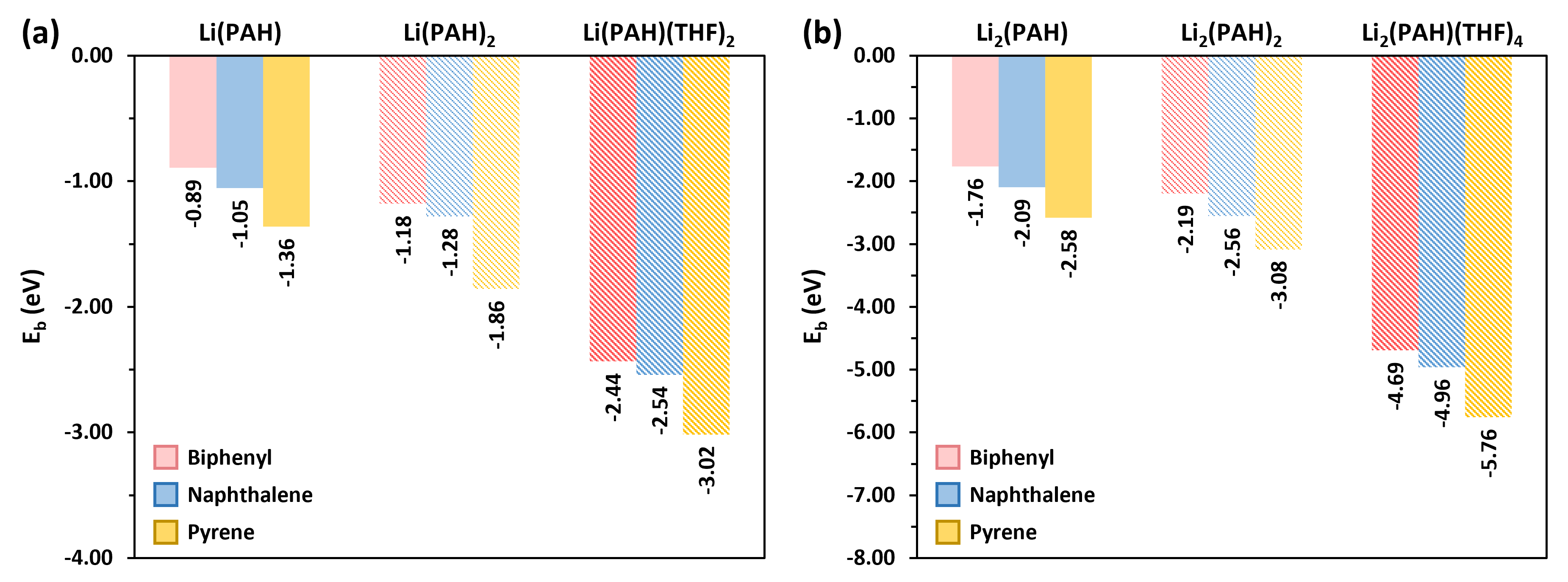


Figure S6. Calculated binding energies (E_b_, in eV) among Li, PAH, and THF in (a) single-lithium and (b) double-lithium systems for three PAHs: biphenyl, naphthalene, and pyrene.


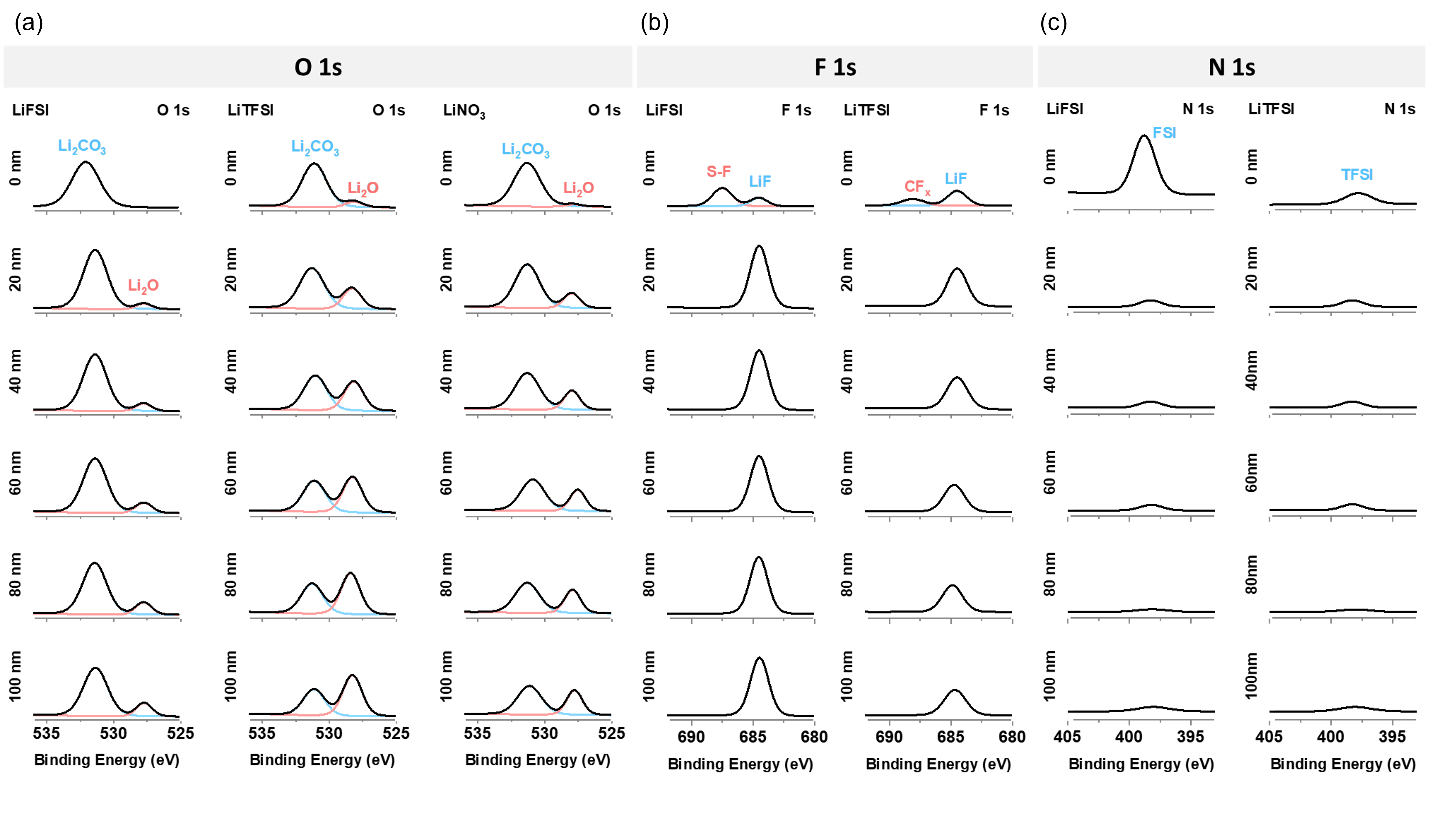


Figure S7. XPS depth profiles of the (a) O 1s, (b) F 1s, and (c) N 1s spectra for lithium surfaces treated with different lithium salt soaking solutions.


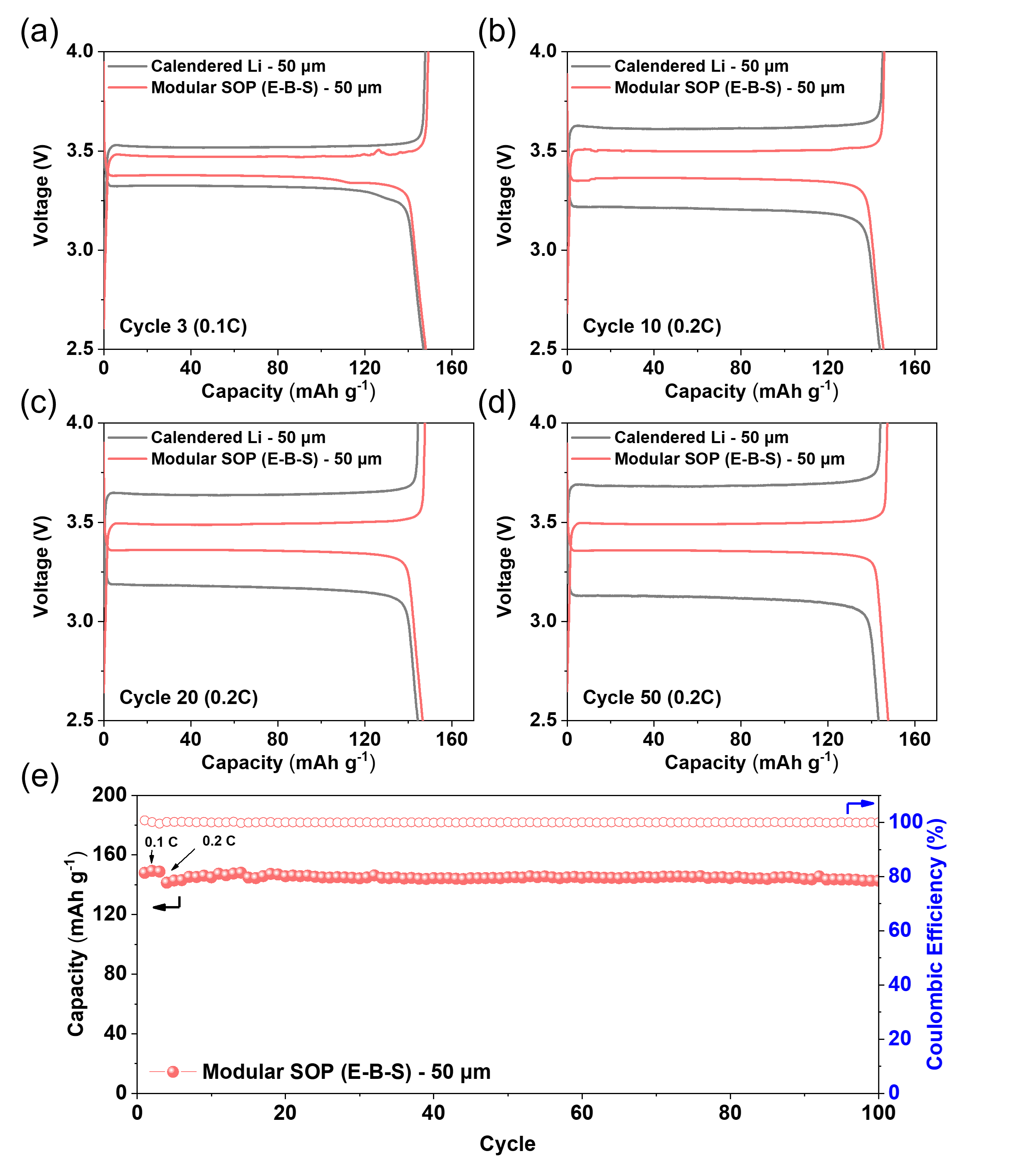


**Figure S8.** Electrochemical evaluation of Li||LFP full cells assembled with 50 μm calendered lithium foils. Representative charge–discharge voltage profiles of full cells using as-received Li and E–B–S-treated Li at the (a) 3^rd^, (b) 10^th^, (c) 20^th^, and (d) 50^th^ cycles. (e) Cycling performance of Li||LFP full cells employing E–B–S-treated 50 μm calendered Li.


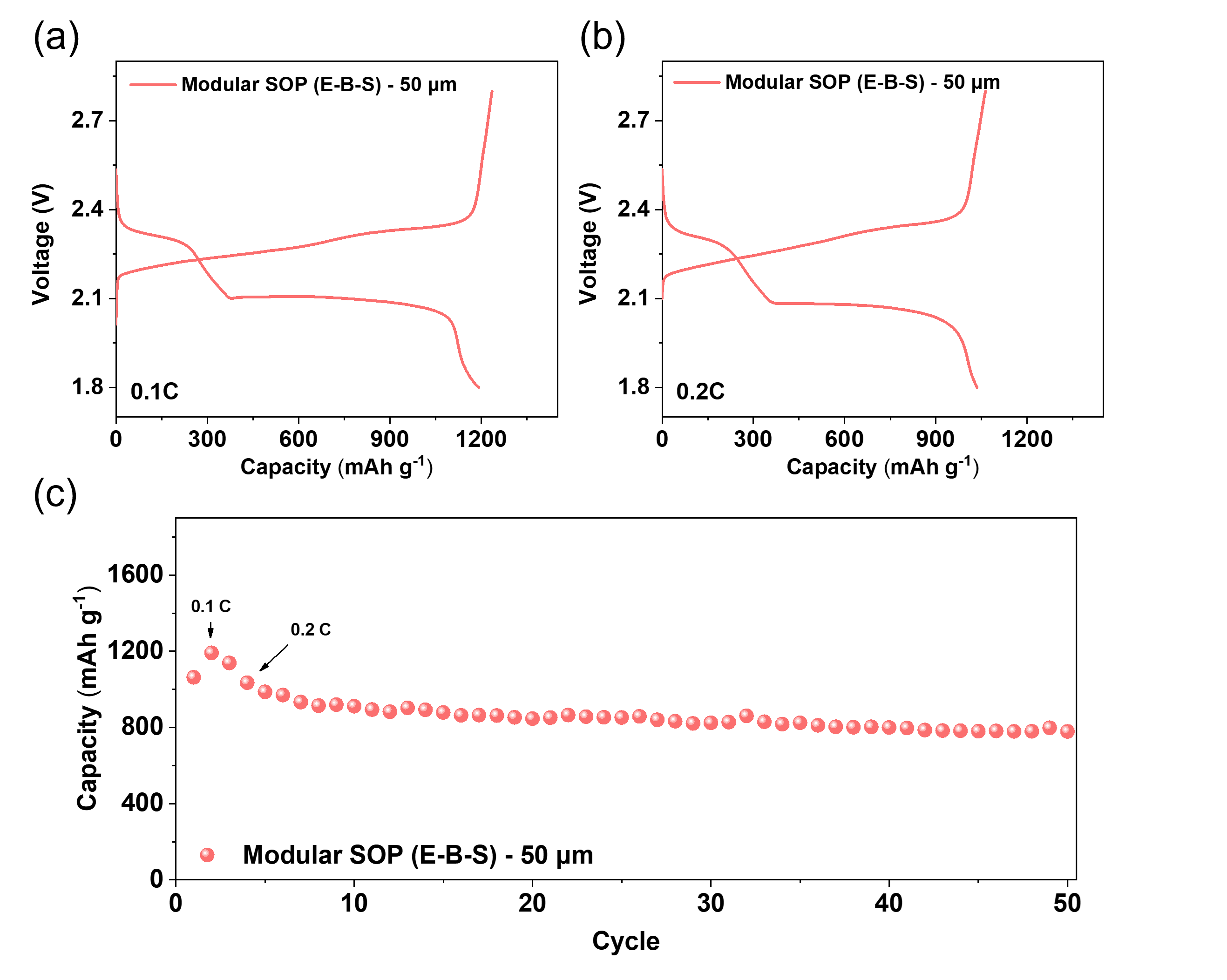


**Figure S9.** Electrochemical evaluation of Li||S full cells assembled with 50 μm E–B–S-treated lithium electrodes. Representative charge–discharge voltage profiles measured at (a) 0.1 C and (b) 0.2 C. (c) Cycling performance of Li||S full cells employing E–B–S-treated lithium.
